# Genome-wide identification of potential odontogenic genes involved in the dental epithelium-mesenchymal interaction during early odontogenesis

**DOI:** 10.1186/s12864-023-09140-8

**Published:** 2023-04-03

**Authors:** Jiawen Chen, Tianyu Sun, Yan You, Binbin Lin, Buling Wu, Jingyi Wu

**Affiliations:** 1 Department of Stomatology, Nanfang Hospital, Southern Medical University, Guangzhou, 510515 Guangdong China; 2 School of Stomatology, Southern Medical University, Guangzhou, 510515 China; 3 Department of Periodontology, Stomatological Hospital, Southern Medical University, Guangzhou, 510280 China; 4 Southern Medical University- Shenzhen Stomatology Hospital (Pingshan), ShenZhen, 518118 China; 5 Center of Oral Implantology, Stomatological Hospital, Southern Medical University, Guangzhou, 510280 China

**Keywords:** Proteoglycans, Glycosaminoglycans, Odontogenesis, Epithelial-mesenchymal interaction, Sulfation

## Abstract

**Background:**

Epithelium-mesenchymal interactions are involved in odontogenic processes. Previous studies have focused on the intracellular signalling regulatory network in tooth development, but the functions of extracellular regulatory molecules have remained unclear. This study aims to explore the gene profile of extracellular proteoglycans and their glycosaminoglycan chains potentially involved in dental epithelium-mesenchymal interactions using high-throughput sequencing to provide new understanding of early odontogenesis.

**Results:**

Whole transcriptome profiles of the mouse dental epithelium and mesenchyme were investigated by RNA sequencing (RNA-seq). A total of 1,281 and 1,582 differentially expressed genes were identified between the dental epithelium and mesenchyme at E11.5 and E13.5, respectively. Enrichment analysis showed that extracellular regions and ECM-receptor interactions were significantly enriched at both E11.5 and E13.5. Polymerase chain reaction analysis confirmed that the extracellular proteoglycan family exhibited distinct changes during epithelium-mesenchymal interactions. Most proteoglycans showed higher transcript levels in the dental mesenchyme, whereas only a few were upregulated in the epithelium at both stages. In addition, 9 proteoglycans showed dynamic expression changes between these two tissue compartments. Gpc4, Sdc2, Spock2, Dcn and Lum were expressed at higher levels in the dental epithelium at E11.5, whereas their expression was significantly higher in the dental mesenchyme at E13.5, which coincides with the odontogenic potential shift. Moreover, the glycosaminoglycan biosynthetic enzymes Ext1, Hs3st1/5, Hs6st2/3, Ndst3 and Sulf1 also exhibited early upregulation in the epithelium but showed markedly higher expression in the mesenchyme after the odontogenic potential shift.

**Conclusion:**

This study reveals the dynamic expression profile of extracellular proteoglycans and their biosynthetic enzymes during the dental epithelium–mesenchymal interaction. This study offers new insight into the roles of extracellular proteoglycans and their distinct sulfation underlying early odontogenesis.

**Supplementary Information:**

The online version contains supplementary material available at 10.1186/s12864-023-09140-8.

## Introduction

Mammalian tooth development has long been used as a representative model for studying the molecular mechanisms underlying organogenesis. The reciprocal inductive interactions between the dental epithelium and the underlying neural crest-derived mesenchyme represent the common pattern for the development of ectodermal placodes deployed in diverse types of epithelium organogenesis, such as salivary glands, lungs, kidneys, mammary glands, hair follicles and limb buds. Conserved signalling pathways such as WNT, BMP, SHH, and FGF are extensively involved in mediating signalling communication during tooth development [1, 2]. Numerous signalling molecules, morphogens and cytokines interact with extracellular components, such as proteoglycans, to transfer and potentiate signal transduction in cells [3, 4]. A delicate balance within these signal transduction pathways is critical for mediating the biological processes of tooth development, including epithelial invagination and mesenchymal condensation [5]. Accumulating evidence shows that proteoglycans are prevalent in the development of mammalian teeth at different stages and are critical signalling regulators during organogenesis [6–8]. However, their roles in mediating odontogenesis, particularly the crosstalk between the dental epithelium and mesenchymal compartments, remain to be elucidated [9].

Proteoglycans are a family of polysaccharide macromolecules composed of core proteins and covalently linked glycosaminoglycan side chains (GAGs). They are widely expressed on cell surfaces and within the ECM of eukaryotic cells [10]. Their expression pattern is finely regulated in different spatial and temporal contexts to mediate various biological and pathological processes, such as organogenesis, tissue development and cancer initiation and progression [11–14].

GAGs are linear polysaccharides composed of repeated disaccharide units. Based on these disaccharide units, GAGs are divided into 5 primary types: hyaluronic acid (HA), chondroitin sulfate (CS), dermatan sulfate (DS), heparan sulfate (HS) and keratan sulfate (KS). Biosynthesis of GAGs is a nontemplate-driven process involving various enzymes that modify the repeated disaccharide units and generate diverse sulfation patterns. The rich structural diversity enables GAGs to provide a wide range of binding sites for a variety of signalling molecules to assist their diffusion, inhibit their binding to receptors, regulate the activity of the signalling pathway bidirectionally, or bind and protect signalling molecules from protease degradation [15].

Recently, studies have identified distinct spatiotemporal proteoglycan expression during tooth morphogenesis and mineralization [16, 17]. Moreover, our previous study found that GAGs mediate a subtle balance of dental epithelial stem cell homeostasis by regulating FGF10/FGFR2b signalling to restrict tooth number in mice in the early stage of odontogenesis [18]. This finding suggested that PGs and GAGs might be essential regulators of the signalling network in the early stage of tooth development to guide dental stem cell fate commitment and epithelial-mesenchymal interactions. However, their roles in early odontogenesis, especially epithelial-mesenchymal interactions, lack sufficient research. The possible roles of individual proteoglycans as well as their sulfation pattern in odontogenesis also remain unclear. A prerequisite to answering these questions is a comprehensive understanding of gene expression profiles during early tooth development. Thus, this study focused on the gene expression profile of proteoglycans in the early stage of odontogenesis. We comprehensively analysed the expression pattern of proteoglycans and their biosynthetic enzymes in two key stages of early tooth development, E11.5 and E13.5, to provide a foundation for further elucidating the mechanism by which proteoglycans affect cell fate determination in early tooth development.

## Methods

### Tissue samples

All animal experiments were approved by the Ruiye model animal (Guangzhou) Biotechnology Co., Ltd Experimental Animal Ethics Committee (SYXK 2020–0218). Eight-weeks old C57BL/6 mice were crossbred to obtain the embryos on different developmental stages. The embryonic age was determined by vaginal plug (E0.5 day) and further confirmed by morphological criteria. Embryos were harvested at E11.5 and E13.5 respectively. The mandibles and tooth germs were dissected from mice embryos from E11.5 and E13.5 embryos respectively. The isolated mandibles and tooth germs were then treated with dispase digestion for 20–30 min at 37℃ to separate the dental epithelium and the mesenchymal as previously described [18–24]. Both incisors and molars were collected in this study. The separated tissues were then placed on ice and collected for extraction of the total RNA. We carried out three biological replicate experiments for each embryonic stage from a total of 10 embryos.

### Transcriptomics

Total RNA of the dental epithelium and mesenchymal tissues were extracted using Trizol reagent (thermofisher, 15,596,018) following the manufacturer's procedure. Total RNA samples were sent to LC-Bio Technology (Hangzhou) for RNA-sequencing and further analysis. Purified RNA samples were subjected to the 2 × 150 bp paired-end sequencing (PE150) on the Illumina Novaseq™ 6000 (LC-Bio Technology CO., Ltd., Hangzhou, China) following the vendor's protocol.

High quality reads were obtained by Cutadapt (https://cutadapt.readthedocs.io/en/stable/, version: cutadapt-1.9) and were compared with the mouse reference genome (ftp://ftp.ensembl.org/pub/release-101/fasta/mus_musculus/dna/).

Genes differential expression analysis was performed by DESeq2 software [25] (http://www.bioconductor.org/packages/release/bioc/html/DESeq2.html) between dental epithelium and mesenchymal groups on E11.5 and E13.5. The significantly differentially expressed genes were selected with the following parameters: |log_2_fc|> = 1 & q < 0.05. Differentially expressed genes were then used for enrichment analysis of GO functions, KEGG pathways and GSEA analysis using the OmicStudio tools at https://www.omicstudio.cn/tool.

### qRT-PCR analysis

cDNA was synthesized using Evo M-MLV Mix Kit with gDNA Clean for qPCR reagent with 1.0 μg total RNA (Accurate Biology, AG11728) according to the manufacturer description. qRT-PCR was performed using Taq Pro Universal SYBR qPCR Master Mix (Vazyme, Q712) with a CFX Connect Real-Time PCR detection system (Bio-Rad). Primers used in the current study are listed in the table below (Table 1). Expression of each gene was normalized to that of GADPH. The results were expressed as relative expression with the 2^–ΔΔCt^ method [26].

**Table 1:** Primers sequences

Gene symbol	Forward Primers (5’-3’)	Reverse Primers (5’-3’)
Gadph	F: TGGAAAGCTGTGGCGTGATG	R: GGTGGAAGAGTGGGAGTTGC
Krt14	F:AGCGGCAAGAGTGAGATTTCT	R: CCTCCAGGTTATTCTCCAGGG
Vim	F: CCGCAGCCTCTATTCCTCAT	R: TCGATGTAGTTGGCAAAGCG
Gpc3	F: TGATGGTTAAGCCTTGCGGT	R: TCTTCATCCCGTTCCTTGCC
Gpc4	F: GCAAAGTGTCCGTGGTGAAC	R: TCATTGCAAACGGTGCTTGG
Sdc2	F: TAGTGCTGCTTCCCCCAAAG	R: AGGGCCAGTATGGCTCTG
Sdc4	F:CTCAGAGCCCAAGGAACTGG	R: CTGCCAGAGCAACTGAGAC
Fmod	F:AGCAGTCCACCTACTACGACC	R: CAGTCGCATTCTTGGGGACA
Spock2	F: ACCCCCGGCAATTTCATGG	R:TGTCTTCCCAGCTCTTGATGTAA
Dcn	F:GAGAAGGGGGCCGATAAAGTT	R: TAGCAAGGTTGTGTCGGGTG
Lum	F: CTCTTGCCTTGGCATTAGTCG	R:GGGGGCAGTTACATTCTGGTG
Fam20b	F: GCGGACAGAAGTTAAGCCTG	R: TATTCCCACCTGGCCAGTTT
Ext1	F: TGGAGGCGTGCAGTTTAGG	R: GAAGCGGGGCCAGAAATGA
Sulf1	F: TGTGTTCCACCGTTCGGTC	R:CACATCCTGGTCGTCAGTGAG
Ndst3	F: TGCTTGCCACCTTTTGTATGG	R:AGCATCGGAAATCATTGTCCTC
Hs3st1	F: CCCAGCTTGTGCATTCCCA	R: TGTGGAACCATTGGATGCTGT
Hs3st5	F:CCTCCTGTATCTAGTTGCCAGA	R:CCAATGATAATGGCTTTGGGGA
Hs6st2	F:GACCTTCCAGGAACTTCCATTAC	R:CATTCACTCAAGTACCGTGACA
Hs6st3	F:GATGAAAGGTTCAACAAGTGGC	R: CGAAGTTGGTGCATGAGCTG

### Statistical analysis

The data were presented as mean ± standard deviation (mean ± SD) of at least 3 samples in all experiments. We used Student’s t-test to determine statistical significance for normally distributed data with SPSS 17.0 software (SPSS, Inc., Chicago, IL, USA). The level of significance was set at *P*-value < 0.05.

## Results

### Workflow of tissue sampling and confirmation

**Fig. 1:**
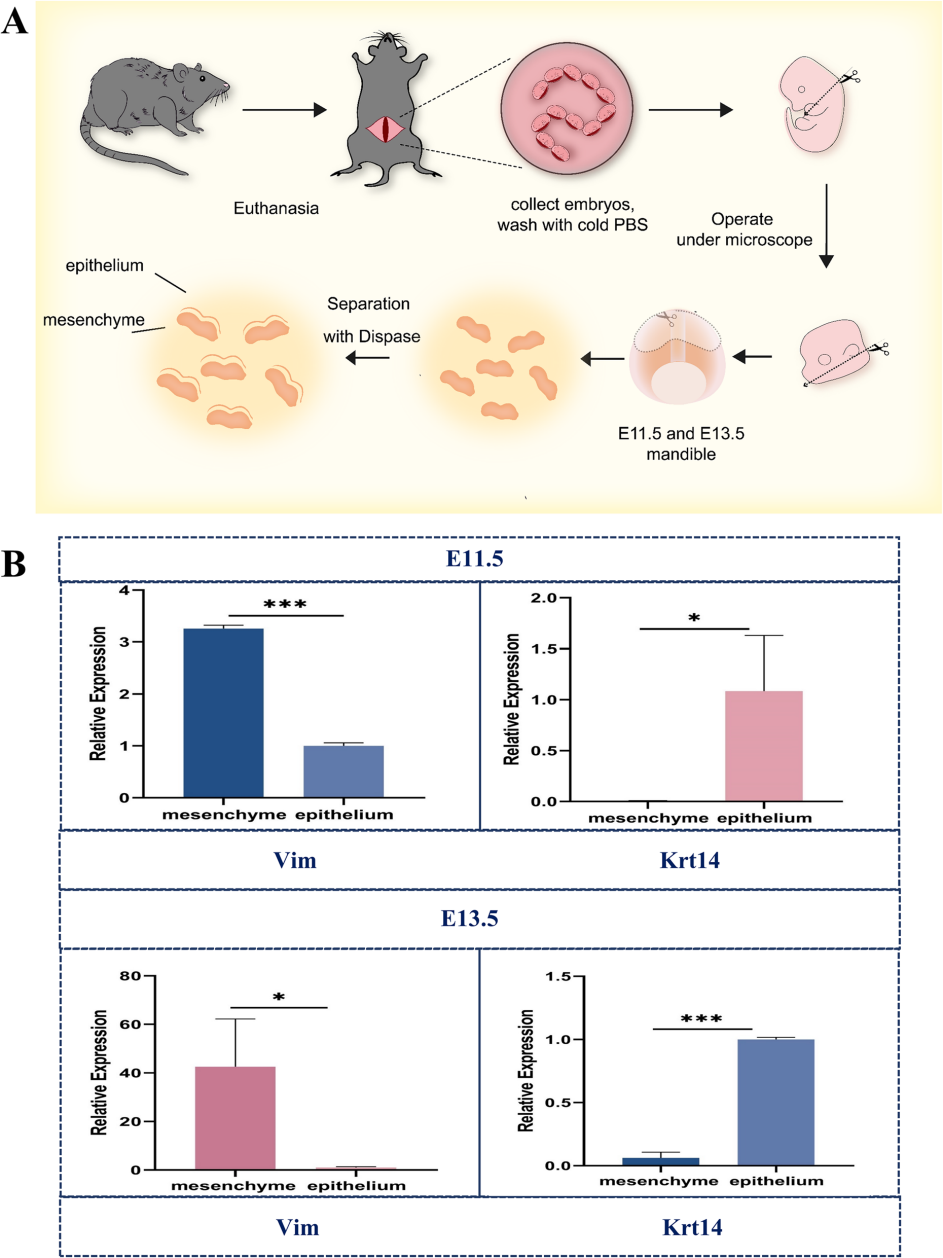
Workflow for sample preparation and validation. **A** Workflow for sample preparation; **B** Expression levels of markers of mesenchymal and epithelial tissue samples from E11.5 and E13.5. (*, *p* < 0.05; ***, *p* < 0.001)

Tissue samples of dental epithelium and mesenchyme were isolated as described in Fig. 1A. After obtaining tissue samples, we performed qRT‒PCR to detect the expression levels of epithelial (Krt14) and mesenchymal (Vim) tissue markers. The transcript level of the dental epithelium-specific gene Krt14 was significantly higher in the epithelial compartments at E11.5 (188-fold higher) and E13.5 (16-fold higher) compared with mesenchymal tissues. The expression of Vim, which encodes a mesenchyme-specific protein, was markedly higher in mesenchymal tissues than in the epithelium at both E11.5 (nearly fivefold higher) and E13.5 (nearly 40-fold higher) (Fig. 1B). These results indicated that the epithelium and mesenchyme tissue were successfully separated.

### Evaluation of transcriptome sequencing data

At least 7.0 Gb of clean data from each sample was obtained from transcriptome sequencing on the Illumina NovaSeq 6000 platform and was available for further expression level analysis after quality control. The Q30 percentages of clean data for all samples were higher than 96.5%, and the GC contents of the clean data for all samples were between 48.5 and 49.5% (supplement file 1). For further analysis, the high-quality clean reads were mapped to the reference Mus musculus genome (v101). Approximately 94.72 to 97.13% of clean reads were successfully mapped to the reference Mus musculus genome.

### Differential gene expression analysis

To investigate changes in the gene expression profiles of embryonic dental epithelium and mesenchyme tissue compartments at different stages, Fragments Per Kilobase of exon model per Million mapped fragments (FPKM) expression values of the genes were calculated based on the read counts. The FC values of each gene were also calculated at different stages between dental mesenchyme and respective dental epithelium. The threshold values FDR ≤ 0.05 and |Log2FC|≥ 1 were used to identify DEGs between groups.

**Fig. 2:**
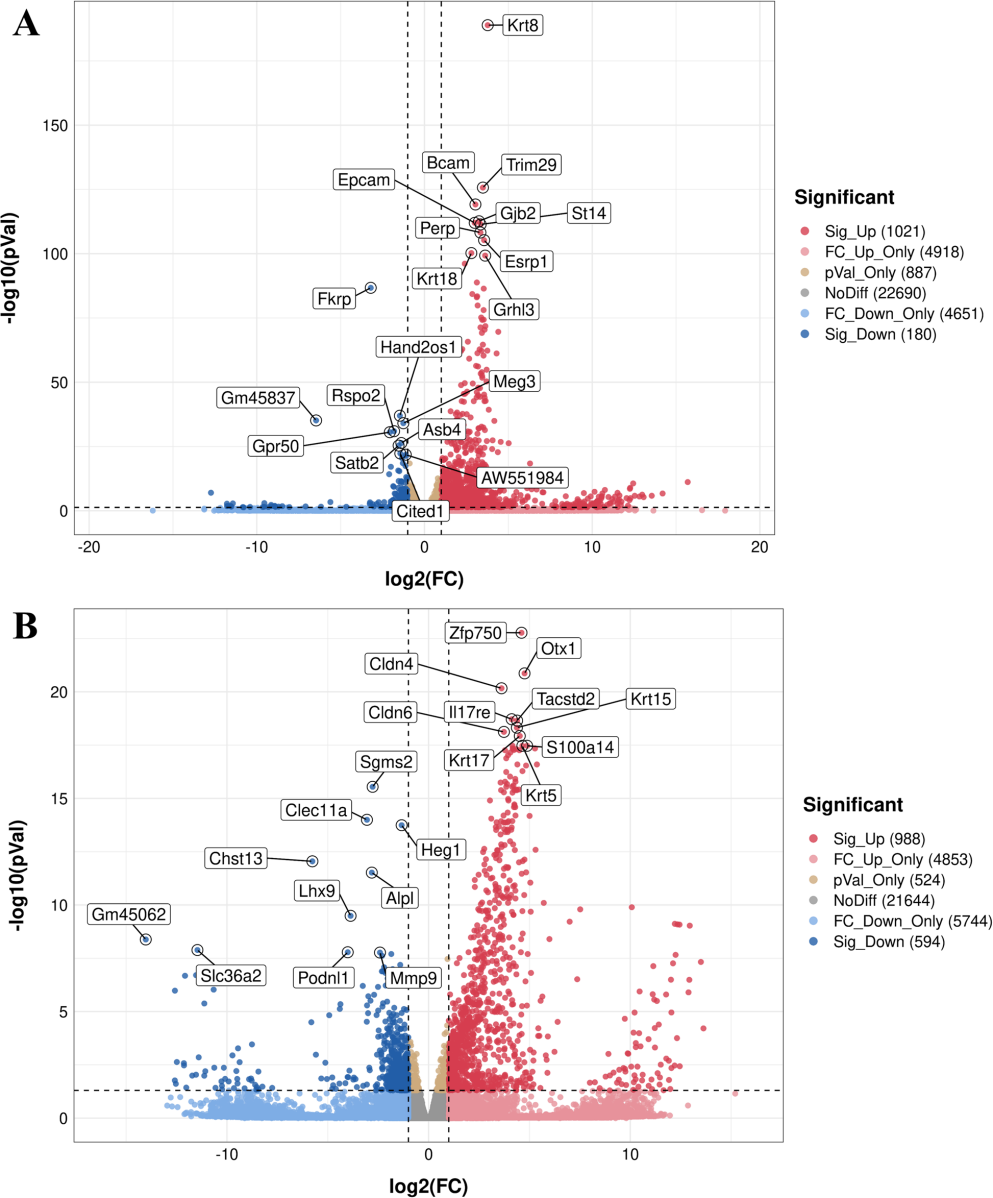
Differentially expressed genes in different groups. **A** Volcano plot for DEGs between the E11 epithelium group and the E11 mesenchymal group. **B** Volcano plot for DEGs between the E13 epithelium group and the E13 mesenchymal group. (DEGs, differentially expressed genes)

Differential expression analysis identified 1,021 upregulated and 180 downregulated genes between E11-E and E11-M and 988 upregulated and 594 downregulated genes between E13-E and E13-M samples (Fig. 2). The top genes selected by both FC and q-value are shown in the graph. At both timepoints, expression of keratin family members, including Krt8, Krt18, Krt15, Krt17 and Krt5, was remarkedly higher in the epithelium. At E13.5, Cldn4 and Cldn6 were more highly expressed in the epithelium, consistent with a previous study [27]. Members of the claudin family have been demonstrated to play significant roles during the later stages of tooth development. Cldn10 is associated with cytodifferentiation of the stratum intermedium, and Cldn3 is essential for amelogenesis [28, 29]. However, whether Cldn4 and Cldn6 also play roles during early odontogenesis remains to be explored. Chst13 is a sulfotransferase involved in CS/DS modification. Chst12 exhibited higher expression in the mesenchymal compartment on E13.5, suggesting that CS/DS modifications might be more active in the mesenchyme than in the epithelium.

### Candidate genes with odontogenic potential in dental epithelium

The odontogenic potential resides in the oral epithelium compartment prior to the bud stage and shifts to the underlying mesenchyme at the early bud stage [5, 30]. Therefore, to identify odontogenic potential genes, we analysed genes with dynamic expression changes between the two compartments during the shift from E11.5 to E13.5.

The DEG results are displayed as volcano plots, and the top 10 GO processes with the largest gene ratios are plotted in order of significantly increased gene ratios in the dental epithelium at E11.5.

**Fig. 3:**
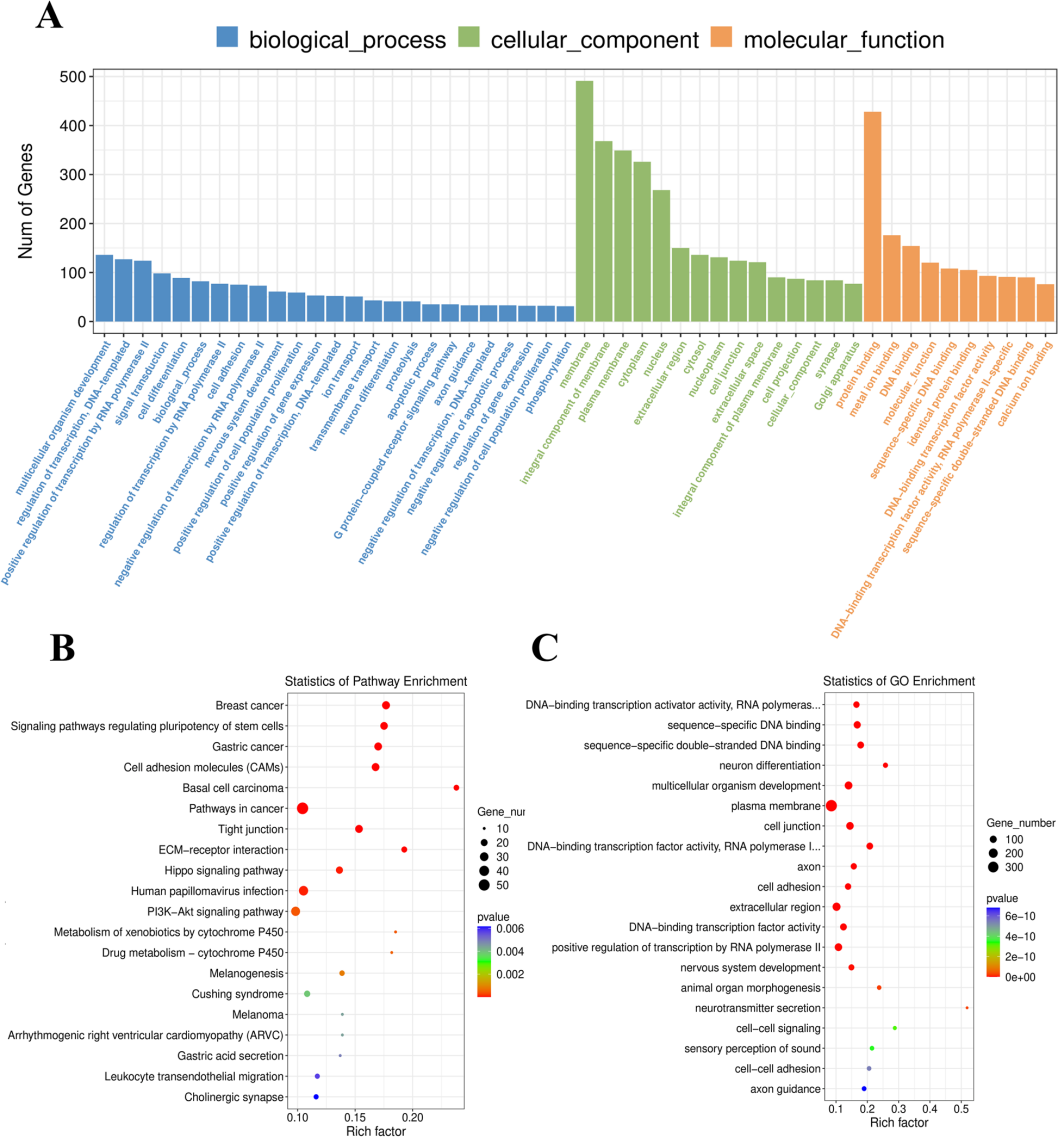
GO analysis and KEGG enrichment analysis of DEGs at E11.5. **A** GO including biological process, cellular components, and molecular function analysis for DEGs; **B**. KEGG pathway analysis for DEGs [31–33]; **C** GO term analysis for DEGs. (DEGs, differentially expressed genes)

The size of the dots represents the number of genes in the significant DEG list associated with the GO term, and the colour represents the p-adjusted value. At E11.5, 1,201 genes were significantly higher in the dental epithelium compared with mesenchyme (Fig. 2A). GO enrichment analysis indicated that 50 GO terms were significantly enriched (Fig. 3A.C). GO terms associated with DEGs at E11.5 were related to the plasma membrane, multicellular organism development and extracellular region (Fig. 3A). The KEGG pathways most enriched were related to pathways in cancer, signalling pathways regulating pluripotency of stem cells and PI3K-Akt signalling (Fig. 3B). GSEA suggested that terms related to ECM were enriched in the dental epithelium group at E11.5 (Additional file S2.A).

### Candidate genes with odontogenic potential in dental mesenchyme

**Fig. 4:**
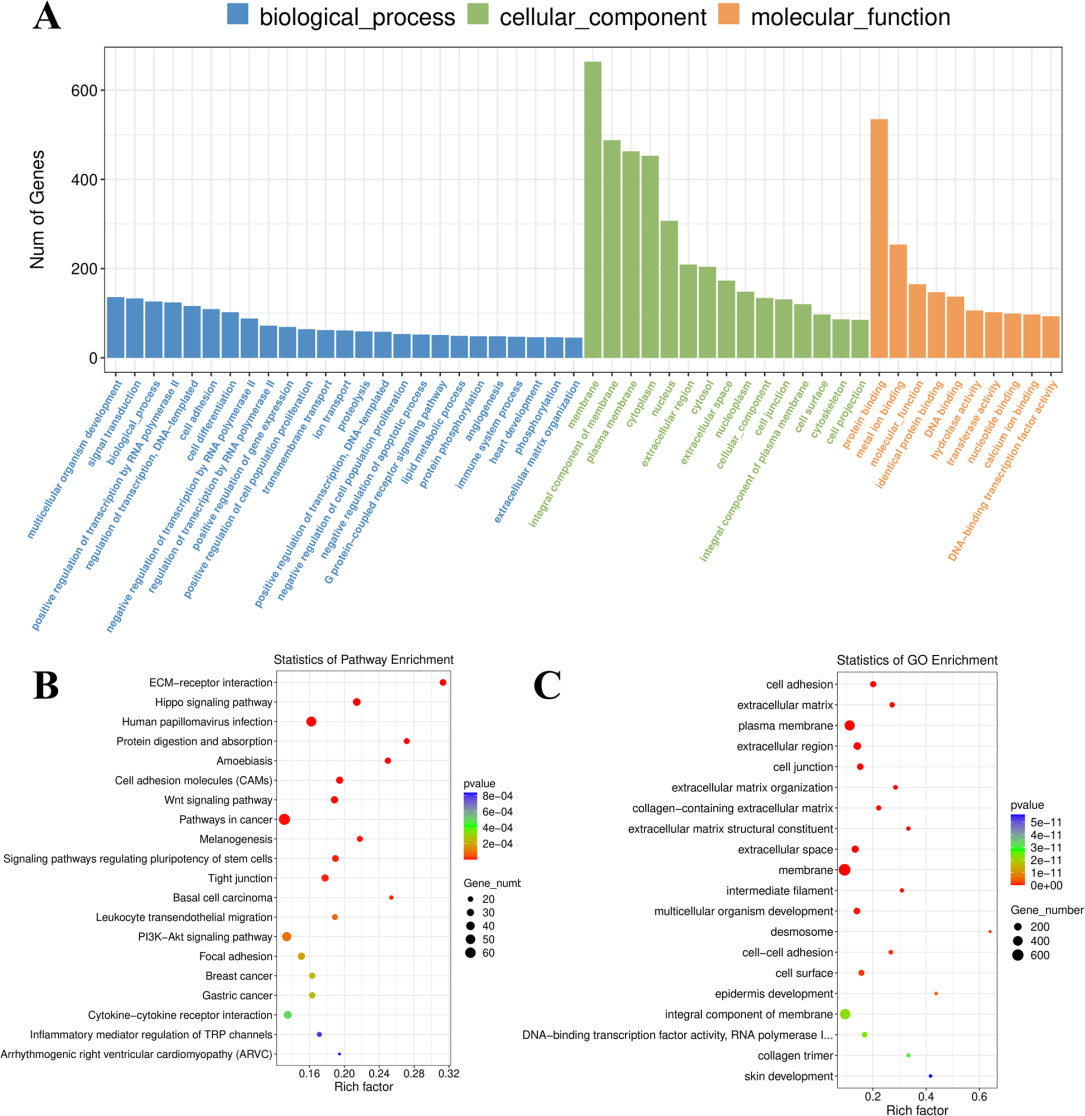
GO analysis and KEGG enrichment analysis of DEGs at E13.5. **A** GO including biological process, cellular components, and molecular function analysis for DEGs; **B** KEGG pathway analysis for differentially expressed genes [31–33]; **C** GO term analysis for DEGs. (DEGs, differentially expressed genes)

A total of 1,582 genes was upregulated in the dental mesenchyme compared with epithelium at E13.5 (Fig. 2B). GO analyses indicated overrepresented GO biological process (GO-BP), GO cellular component (GO-CC), and GO molecular function (GO-MF). According to the GO functional enrichment results, the most enriched terms of these three aspects are shown in Fig. 4A.

GO terms associated with increased DEGs in the E13.5 dental mesenchyme were related to membrane, plasma membrane and extracellular region (Fig. 4C). The KEGG pathways most enriched were related to pathways in cancer, PI3K-Akt signalling, Wnt signalling and ECM-receptor interaction (Fig. 4B). GSEA suggested that terms related to ECM, proteoglycans and proteoglycan biosynthesis were enriched in the dental mesenchymal group on E13.5 (Additional file S2.B).

### Candidate genes with temporal shift expression

**Fig. 5:**
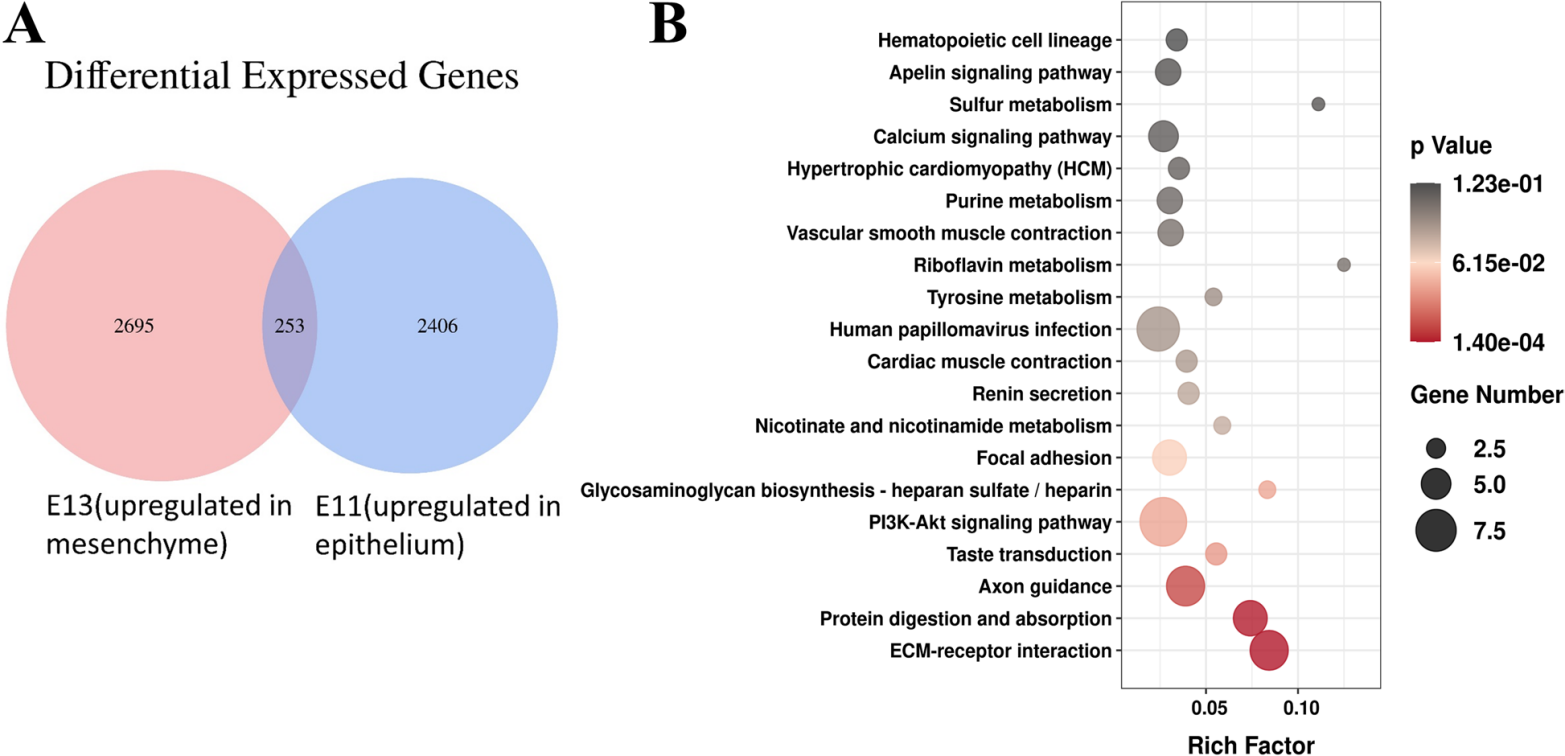
Venn graph, and KEGG enrichment analysis of DEGs with dynamic upregulation in the dental epithelium at E11.5 and in the mesenchyme at E13.5. **A** Venn diagram for intersecting DEGs; **B** KEGG pathway analysis for intersected DEGs [31–33]

To identify genes with odontogenic potential, we examined genes that were more highly expressed in dental epithelium at E11.5 but more highly expressed in mesenchyme at E13.5. We failed to obtain enough genes in this intersection using the strict threshold for DEGs in the analysis outlined above. To gain a more comprehensive analysis, we reanalysed data from E11.5 and E13.5 and identified DEGs with a relaxed threshold (log2fc ≠ 0, *p* < 0.05). We identified 2,659 upregulated DEGs in the epithelium on E11.5 and 2,948 upregulated DEGs in the mesenchyme on E13.5 with 253 intersection genes (Fig. 5A).

We found 253 genes with specific expression profiles reflecting the odontogenic potential shift.

**Fig. 6:**
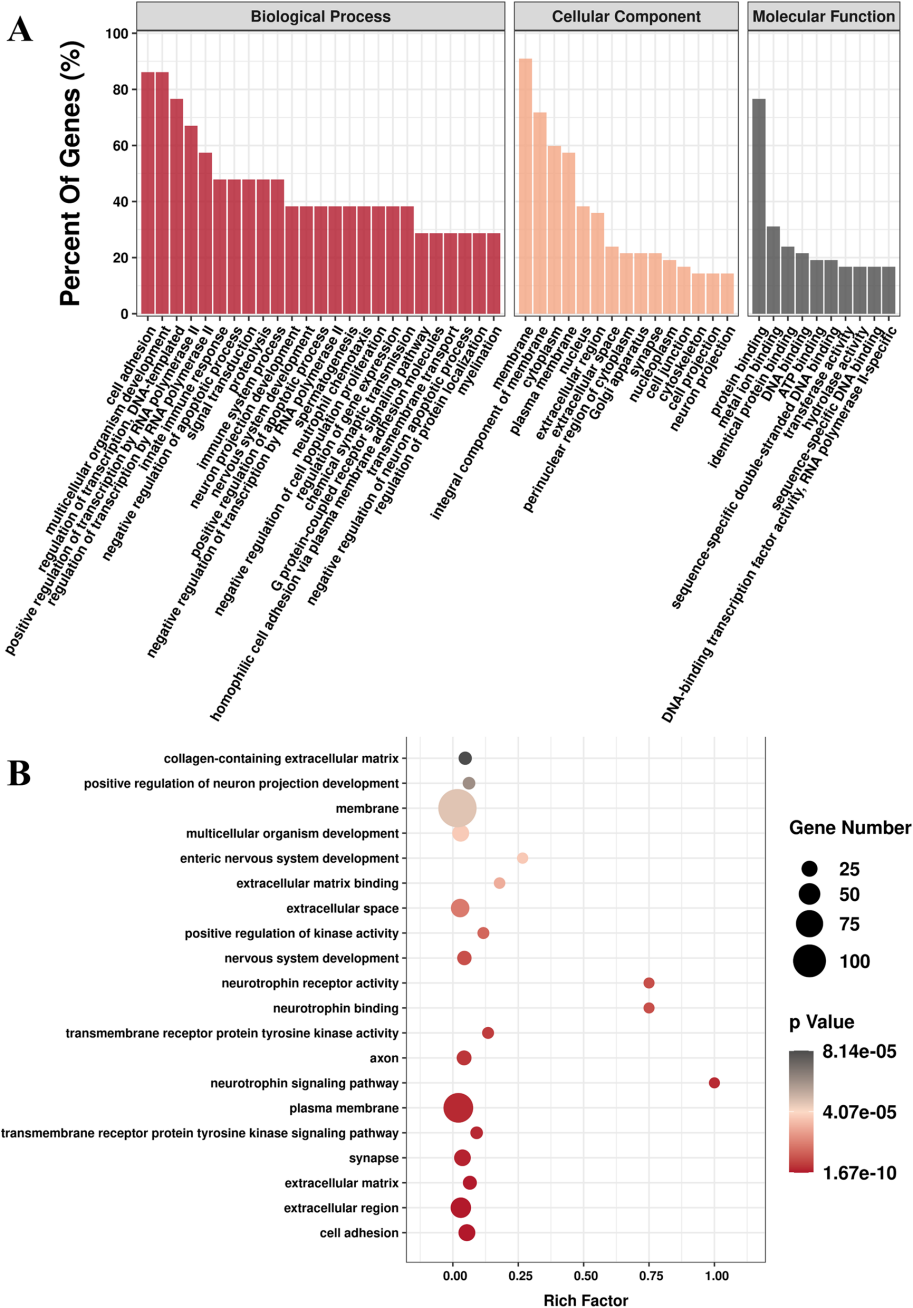
**A**-**B**. GO analysis including biological process, cellular components, and molecular function analysis for intersected DEGs; (DEGs, differentially expressed genes)

We performed GO enrichment analysis on these DEGs to understand how these DEGs functioned during odontogenic processes. The most indicated GO terms were protein binding, plasma membrane and extracellular region (Fig. 6A). This result was similar to the indicated GO terms at E11.5 and E13.5. Pathway analysis was applied to find significant pathways represented by the DEGs. Most of the genes were involved in the PI3K-Akt signalling pathway, ECM-receptor interaction, and pathways in cancer (Fig. 6B).

### Expression profile of proteoglycans in the dental epithelium and mesenchyme

The results of GO analysis showed that the GO term extracellular region was enriched at both E11.5 and E13.5, suggesting that some proteins or molecules in the extracellular region might be involved in the biological process of dental epithelium-mesenchymal interaction during the early stage. In addition, KEGG results indicated that ECM-receptor interactions were significantly enriched, thereby implying that specific proteins in the ECM with potential functions as receptors or coreceptors might be essential for dental epithelium-mesenchymal interactions.

**Fig. 7:**
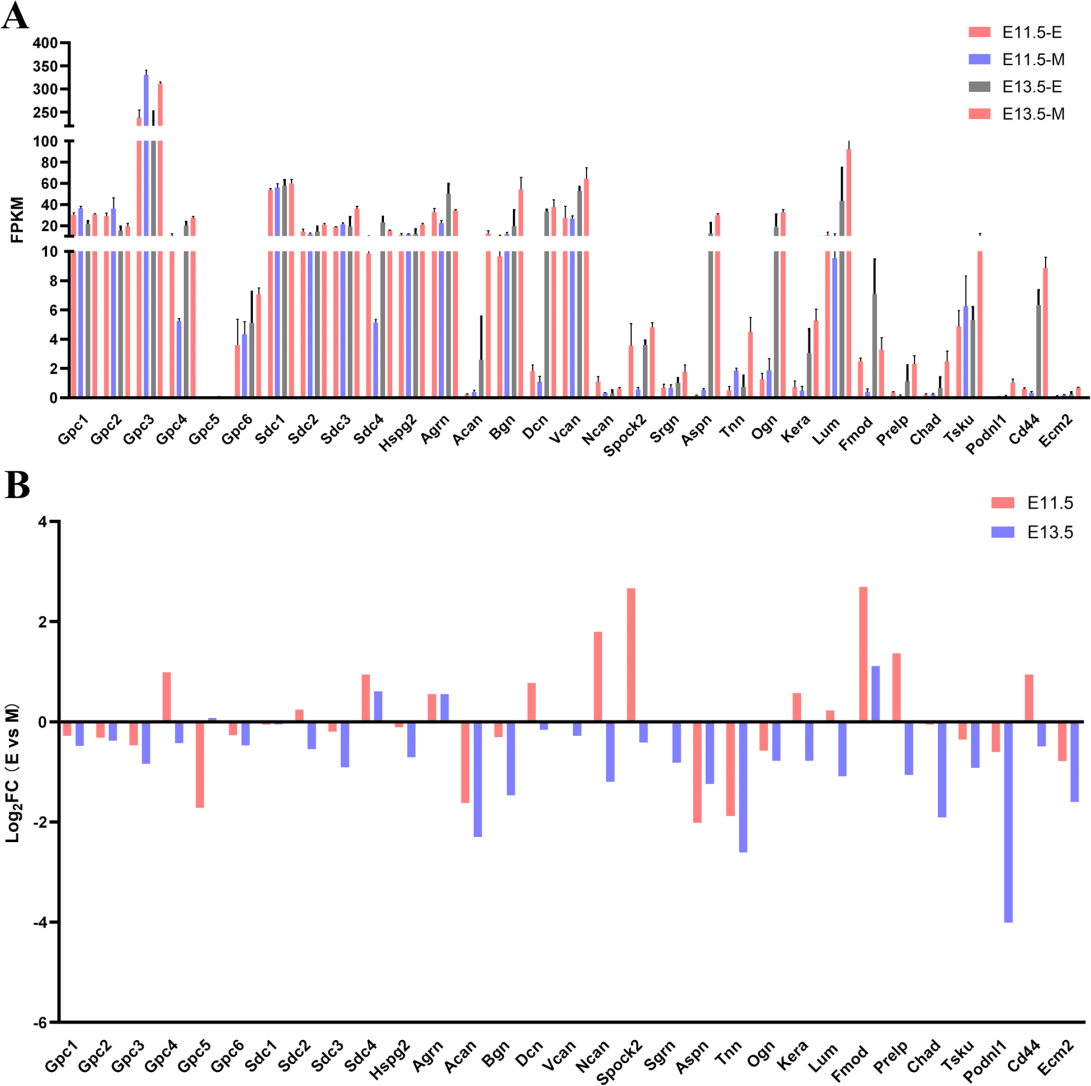
Expression profile of proteoglycans at E11.5 and E13.5 in different compartments. **A** FPKM analysis of proteoglycans at E11.5 and E13.5 in both the dental epithelium and mesenchyme; **B** Log_2_ fold change analysis of proteoglycans

**Fig. 8:**
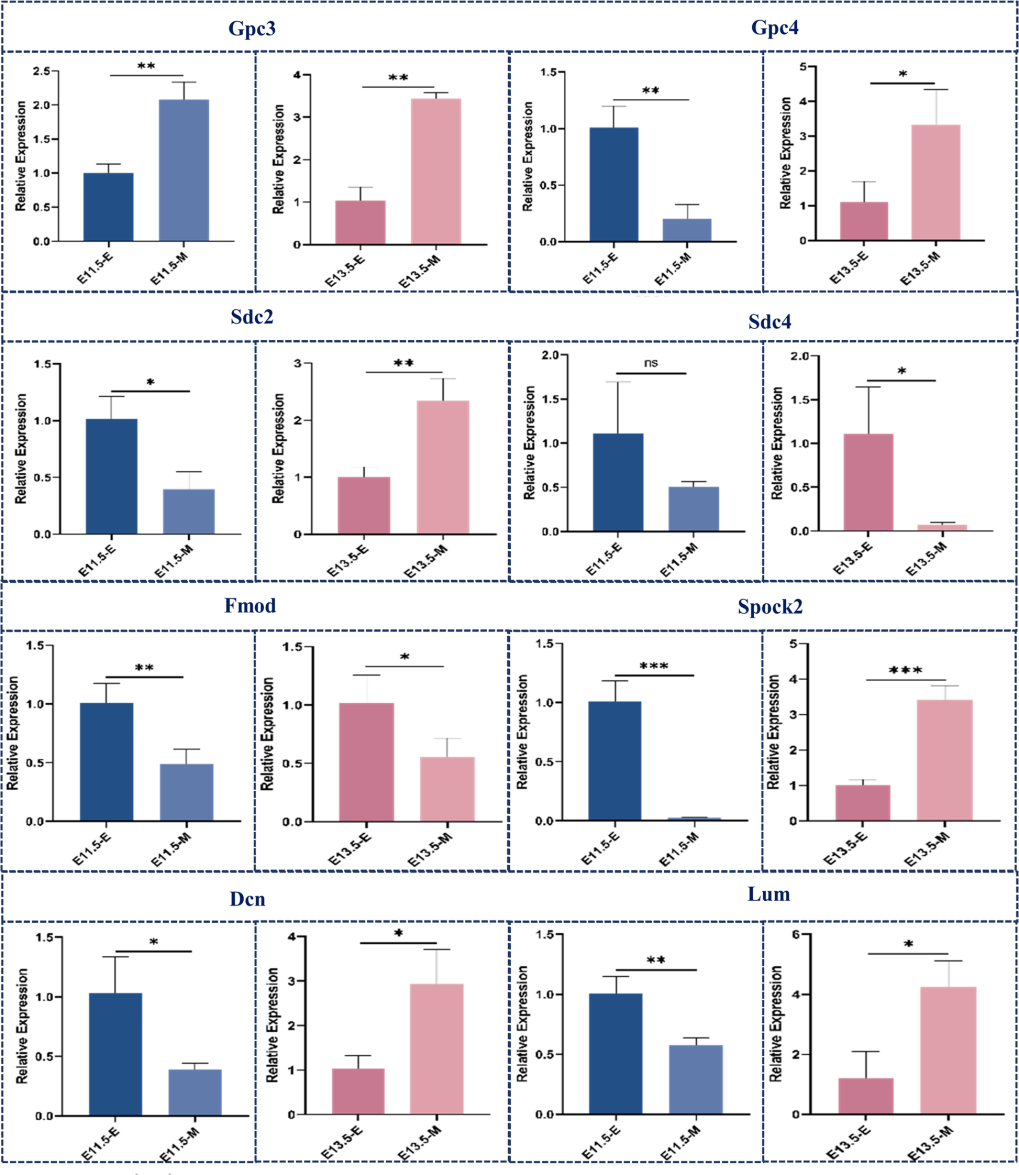
qRT‒PCR analysis of proteoglycans in the dental epithelium and mesenchyme at E11.5 and E13.5. (E, Epithelium; M, Mesenchyme) (*, *p* < 0.05; **, *p* < 0.01; ***, *p* < 0.001)

To understand the expression profile of proteoglycans in the early stage of odontogenesis and provide more clues for understanding their roles during the whole process of tooth development, we performed an analysis of the FPKM of proteoglycans and identified differentially expressed proteoglycans (Fig. 7A). The log_2_FC values for various proteoglycans were used to construct a heatmap and visualize the expression profile (Fig. 7B and additional file S3.A). Gpc4, Sdc-2, Dcn, Lum, Spock2, Ncan, Kera, Prelp and Cd44 were upregulated in the dental epithelium at E11.5 and upregulated in the dental mesenchyme at E13.5, with temporal and spatial shifts of expression between these two tissue compartments. Such spatiotemporal expression implied potential roles for these PGs in guiding odontogenic competence between dental epithelium and mesenchyme and in signal transduction between these two compartments. Moreover, we performed qRT‒PCR to further validate their expression (Fig. 8). Gpc4 expression was higher in the dental epithelium than mesenchyme (4.7-fold) at E11.5 and was upregulated in the mesenchyme (nearly 5.0-fold) at E13.5. The expression of Sdc2 was twice as high in the dental epithelium than in the mesenchyme at E11.5, but it was 2.2-fold higher in the dental mesenchyme than in the epithelium at E13.5. In addition, the transcript level of Spock2 at E11.5 was 38-fold higher in the dental epithelium but 3.5-fold higher in the dental mesenchyme at E13.5. Other proteoglycans with dynamic expression profiles included Dcn and Lum. The transcript level of Dcn was twofold higher in the dental epithelium than in the mesenchyme at E11.5, whereas it was upregulated threefold in the mesenchyme at E13.5. The expression of Lum in the E11.5 epithelial compartments was twice as high as that in mesenchyme, whereas upregulation in dental mesenchyme by E13.5 established a nearly 4-fold higher expression level compared with epithelial tissue (Fig. 8).

In addition to this dynamic expression pattern, the following two other proteoglycans expression profiles were observed: PG transcript levels were significantly higher in the dental epithelial compartment at both E11.5 and E13.5, or PG transcript levels were significantly higher in the dental mesenchyme at both E11.3 and E13.5. Sdc4 and Fmod were significantly higher in the dental epithelial compartment at both E11.5 and E13.5. Based on the preliminary results of RNA-seq, we performed qPCR to obtain a more quantitative expression level. In qRT‒PCR analysis, Sdc4 was 2.0-fold more highly expressed in dental epithelial tissue at E11.5 and nearly 16-fold more highly expressed at E13.5. The expression of Fmod was nearly 2.0-fold higher in the dental epithelium at E11.5 and E13.5. For proteoglycans upregulated in the dental mesenchymal tissue, Gpc3 was expressed at nearly 2.0-fold higher and 3.0-fold higher levels in the dental mesenchymal tissue at E11.5 and E13.5, respectively.

**Fig. 9:**
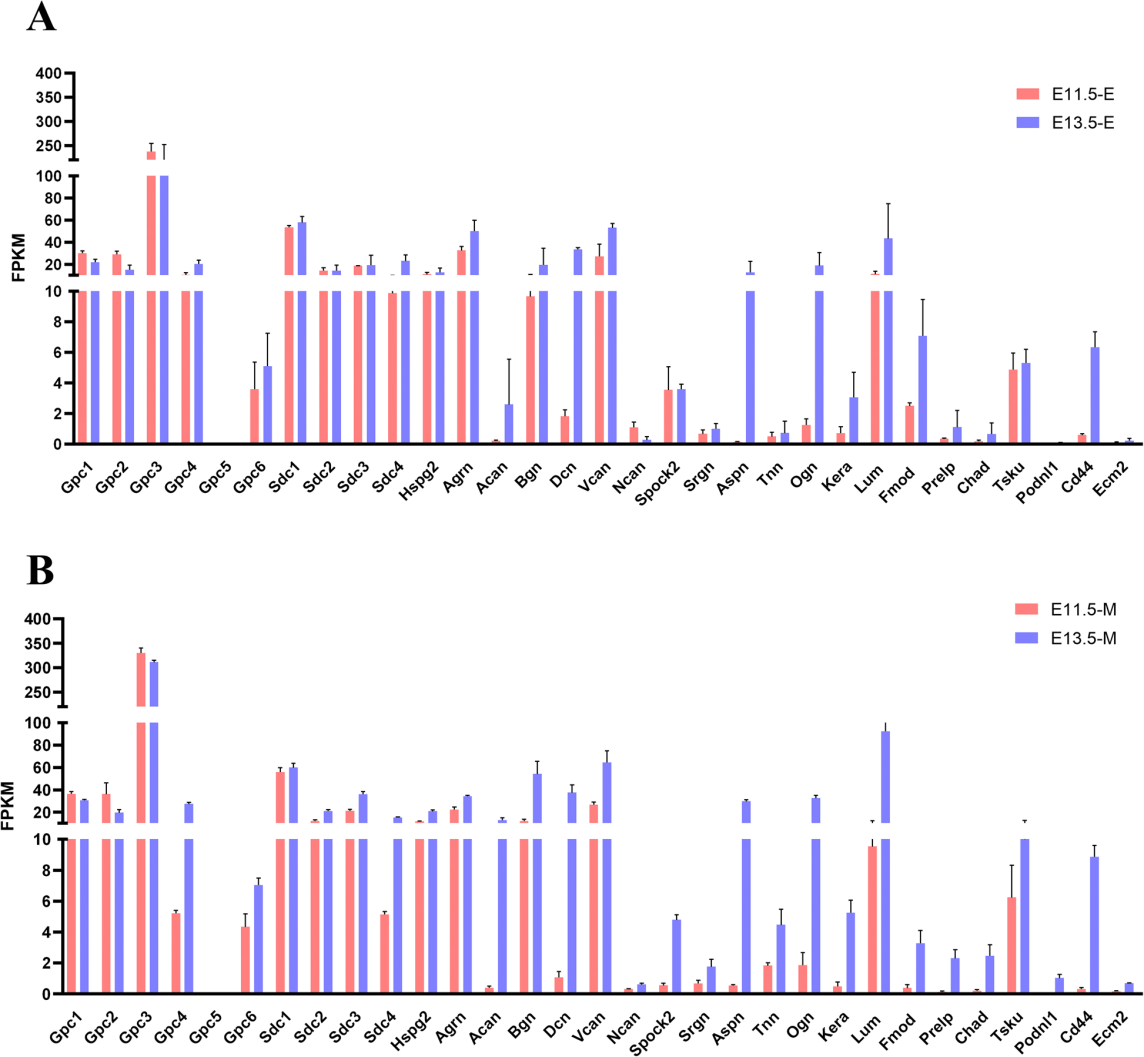
Expression profile of proteoglycans in the dental epithelium and mesenchyme at E11.5 and E13.5. **A** Expression profile of proteoglycans in dental epithelium at E11.5 and E13.5. **B** Expression profile of proteoglycans in dental mesenchyme at E11.5 and E13.5

From a developmental perspective, most PGs exhibited higher transcript levels in the mesenchymal compartments from E11.5 to E13.5. Among these PGs, Gpc4, Gpc6, Sdc4, Acan, Bgn and Dcn showed significantly higher expression levels in the dental mesenchyme at E13.5 compared with E11.5 (Fig. 9B).

For dental epithelial tissue, only Gpc1 and Gpc2 showed slightly lower expression at E13.5 than E11.5. Other PGs also exhibited higher expression levels as development progressed (Fig. 9A).

Interestingly, this result suggests that the temporal shift from dental epithelium to mesenchyme might be induced by higher expression growth of PGs in the mesenchyme compared with epithelium rather than an “on” and “off” expression pattern.

### Expression profile of proteoglycan biosynthetic enzymes in the dental epithelium and mesenchyme

**Fig. 10:**
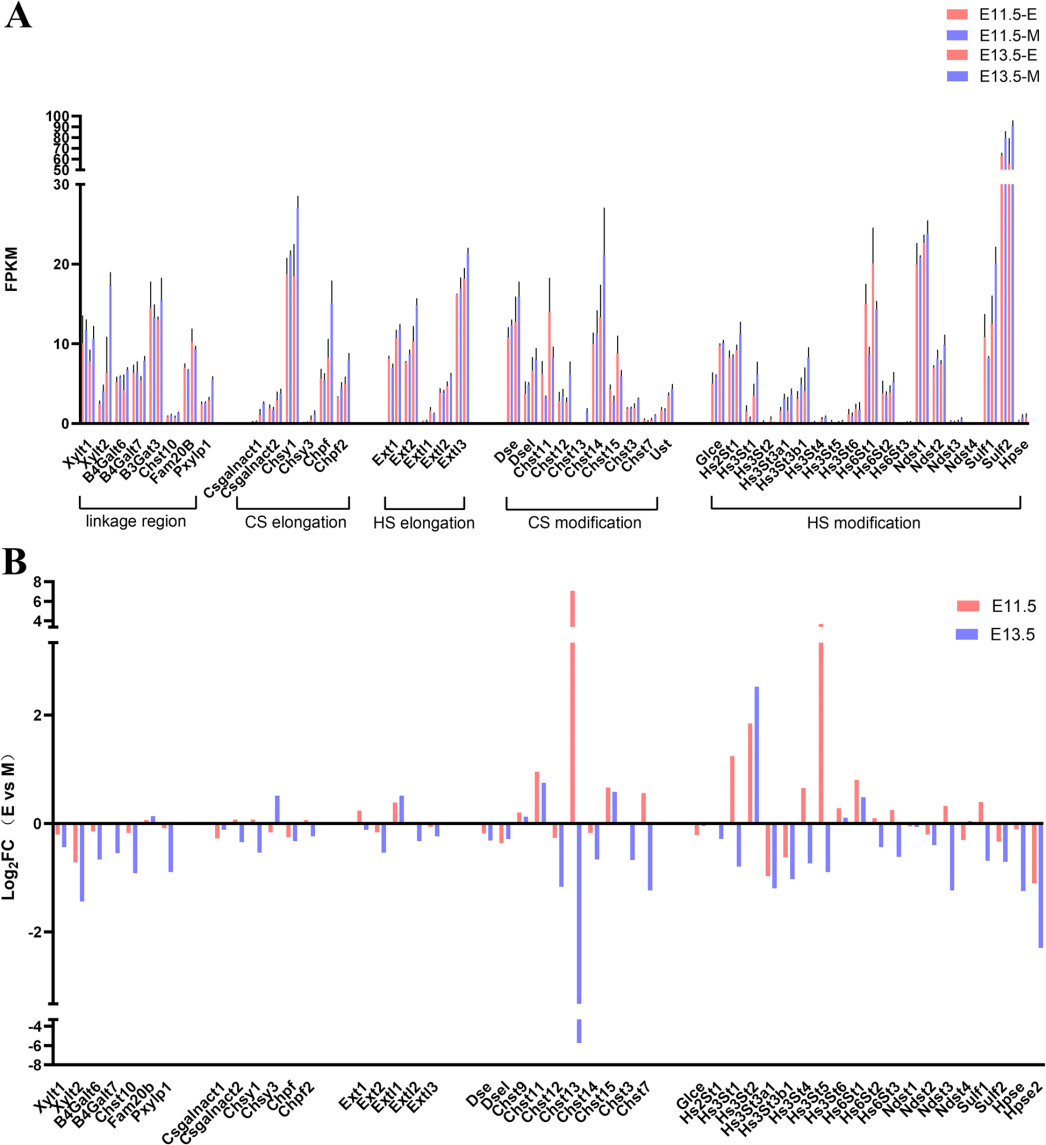
Expression profile of proteoglycan biosynthetic enzymes in the dental epithelium and mesenchyme at E11.5 and E13.5. **A** FPKM analysis of proteoglycan biosynthetic enzymes at E11.5 and E13.5 in both the dental epithelium and mesenchyme; **B** Log2-fold change analysis of proteoglycan biosynthetic enzymes

Glycosaminoglycans are essential to both the structure and biological functions of proteoglycans. Thus, we also explored the expression profile of glycosaminoglycan biosynthetic enzymes (Fig. 10).

Enzymes involved in the common linkage region include xylosyl transferase (Xylt1 and Xylt2), β4-galactoslytransferase (β4galt7), β4-galactoslytransferase (β4galt6) and β3-galactoslytransferase (β3gat1, β3gat2, β3gat3).

**Fig. 11:**
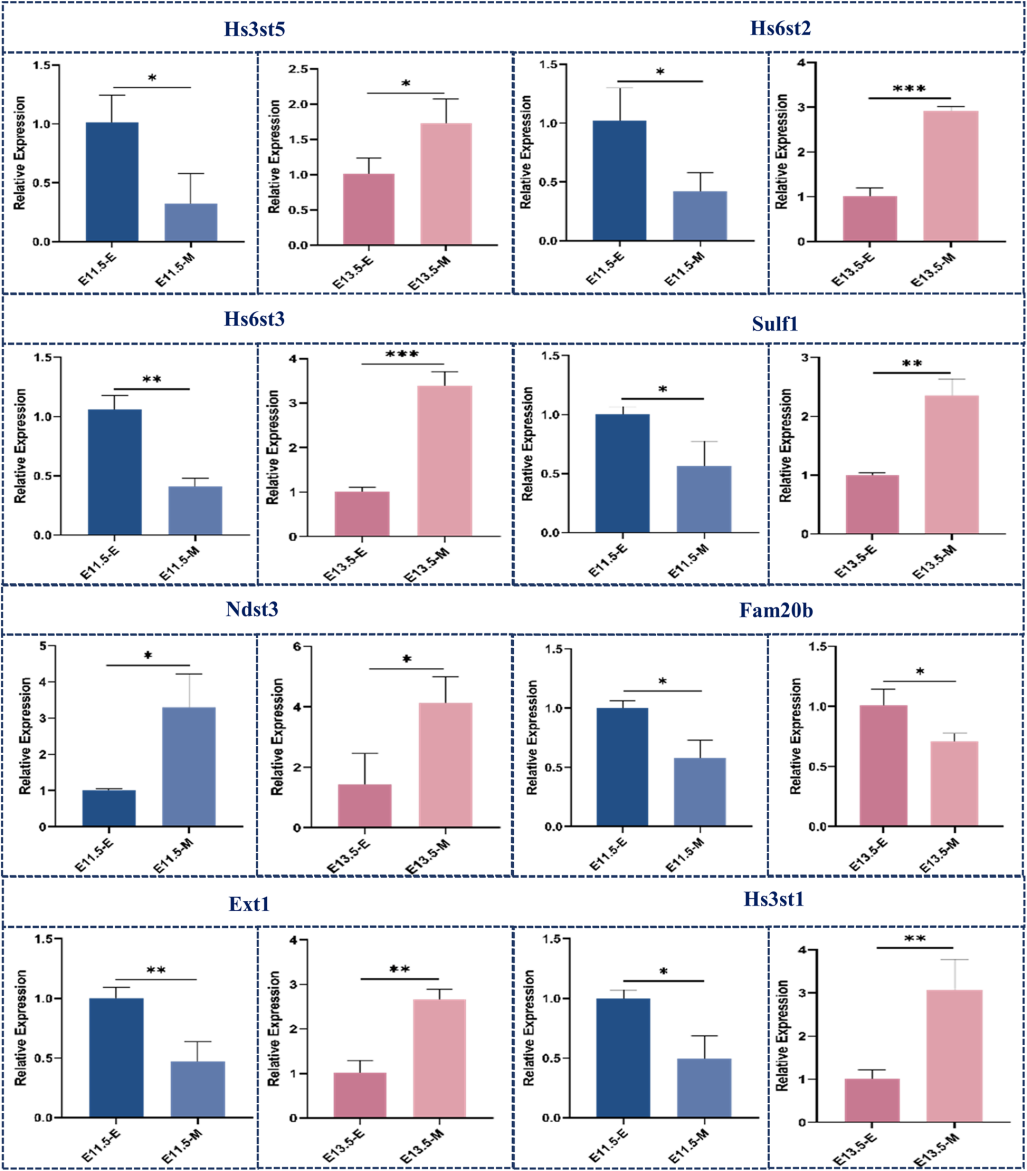
qRT‒PCR analysis of proteoglycans biosynthetic enzymes in the dental epithelium and mesenchyme at E11.5 and E13.5. (E, Epithelium; M, Mesenchyme) (*, *p* < 0.05; **, *p* < 0.01; ***, *p* < 0.001)

Most of these enzymes exhibited similar expression patterns, including Xylt1, Xylt2, B4galt6, B4galt7, Chst10 and Pxylp1. These enzymes were highly expressed in the dental mesenchyme at both E11.5 and E13.5. Among these enzymes, only Fam20b was more highly expressed in the dental epithelium compared with mesenchyme at both stages (Fig. 11).

HS elongation enzymes include glucuronosyltransferase (Ext1 and Ext2) and α4-GlcNAc transferase (Extl2 and Extl3). The transcript level of Ext1 was higher in the dental epithelium at E11.5, but it was upregulated in the mesenchyme at E13.5. In contrast, the expression levels of Ext2, Extl2 and Extl3 were higher in the dental epithelium at both developmental stages. Only Extl1 was upregulated in the mesenchyme at both stages.

Elongation of CS/DS chains requires N-acetylgalactosaminyltransferase-1 (CSGalNAcT-1), N-acetylgalactosaminyltransferase-2 (CSGalNAcT-2), and the chondroitin polymerase complex (encoded by Chsy-1/Chsy-2/Chsy-3/Chpf). The expression of Csgalnact1 and Chpf exhibited similar expression profiles; both were upregulated in the mesenchyme during odontogenesis. Chsy1 and Chpf2 were upregulated in the epithelium at E11.5 and upregulated in the mesenchyme at E13.5. In contrast, chsy3 was downregulated at E11.5 but was upregulated at E13.5 in the dental epithelium.

HS N-deacetylase, N-sulfotransferase isoforms 1–4 (Ndst1, Ndst2, Ndst3, and Ndst4), HS 5ʹ-uronosyl epimerase (Glce), HS 2-O-sulfotransferase (Hs2st1), HS 6-O-sulfotransferase isoforms 1–3 (Hs6st1, Hs6st2, Hs6st3), and HS 3-O-sulfotransferase isoforms (Hs3st1, Hs3st2, Hs3st3a1, Hs3st3b1, Hs3st5, and Hs3st6) are involved in the modification of the HS GAG repeating unit. Among them, the transcript levels of Hs3st1, Hs3st4, Hs3st5, Hs6st2, Hs6st3, Ndst2 and Sulf1 were higher in the dental epithelium at E11.5, but they were upregulated in the mesenchyme at E13.5. In contrast, the expression levels of Hs3sta1, Hs3stb1, Sulf2, Hpse and Hpse2 were higher in the dental mesenchyme in the two developmental stages. Only Hs3st2 was upregulated in the mesenchyme at both stages.

CS/DS are sulfated by various sulfotransferases. 4-O-sulfation is catalysed by chondroitin 4-O-sulfotransferase (C4ST). The addition of a sulfate group to the 6-O position of GalNAc is accomplished by chondroitin 6-O-sulfotransferase (C6ST). GalNAc 4-sulfate 6-O-sulfotransferase (GalNAc4S-6ST) can also transfer sulfate groups to both the 4-O and 6-O positions of GalNAc. Transfer of the sulfate group to the 2-O position of GlcNAc is accomplished by uronyl 2-O-sulfotransferase (UST). For DS chains, epimerization of GlcA to IdoA is catalysed by GlcA C-5 epimerase (DS-epimerase). The repeating unit of DS can be modified by dermatan 4-O-sulfotransferase (D4ST) transferring the sulfate group to the 4-O position of GalNAc. The expression of Dse, Dsel and Chst12 was lower in the epithelium at both E11.5 and E13.5, whereas chst9, Chst11 and Chst15 were higher in the epithelium at both stages. Among these enzymes, Chst13 and Chst7 were more highly expressed in the epithelium at E11.5 and upregulated in the mesenchyme at E13.5.

### Quantitative real-time PCR (RT‒qPCR) analysis

**Fig. 12:**
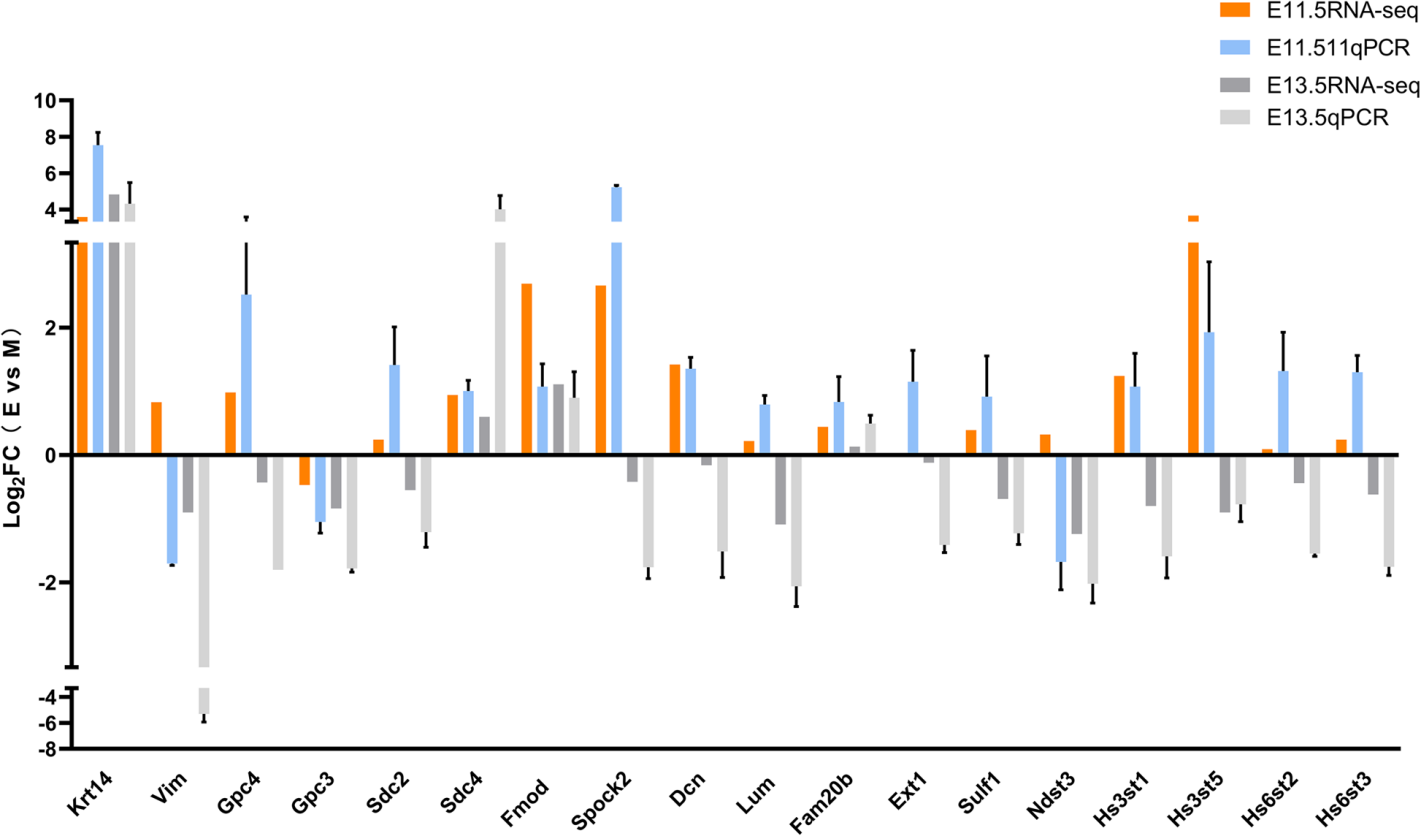
qRT‒PCR validation of RNA-sequencing results

We selected ten genes, Krt14, Vim, Gpc3, Gpc4, Sdc2, Sdc4, Fmod, Spock2, Dcn and Lum, for RT‒qPCR analysis to validate DEGs identified from RNA sequencing (Fig. 12). As expected, the results showed that these genes were mostly consistently upregulated or downregulated with the gene expression changes based on RNA-Seq, indicating that the DEGs obtained from RNA-sequencing were reliable.

## Discussion

Tooth development is an ordered process of mutual induction between odontogenic epithelium and mesenchyme, in which odontogenic stem cells differentiate and form teeth in accordance with developmental procedures. This process is regulated by a complex signal transduction network. Each stage of tooth development is regulated by the delicate and complex signal network between cells, which depends on the precise spatial–temporal expression of each signal to guide odontogenesis, cytodifferentiation and development. Many signalling pathways involved in tooth formation have been elucidated. However, the precise molecular mechanisms that mediate interactions between the epithelium and mesenchymal cells remain unclear. PGs are ubiquitously expressed on the cell membrane and in the extracellular matrix. These glycoproteins exert critical roles in facilitating ligand‒receptor interactions and shaping diffusion gradients through the GAG chains attached.

Previous studies have shown that several PGs are actively involved in the embryonic development of multiple tissues and organs. We recently found that proteoglycans have important roles in maintaining the signalling balance that governs murine tooth number [18]. Moreover, by examining the expression pattern of 23 proteoglycans in different regions of murine embryonic teeth, we identified a distinct spatiotemporal expression pattern of proteoglycans in tooth morphogenesis [8]. However, we have not determined a specific type of proteoglycan or the key sulfation group of glycosaminoglycans involved in the regulation of odontogenesis. Therefore, in this study, we further investigated the dynamic expression profile of proteoglycans and their biosynthetic enzymes during early odontogenesis by high-throughput sequencing.

The odontogenic potential represents the ability of one tissue to induce related gene expression in the adjacent tissue to initiate and promote tooth development. On E11.5, odontogenic competence initially resides in the dental epithelium and then shifts to the adjacent mesenchymal tissue on E12.5 [30, 34]. Thus, we chose E11.5 and E13.5 as key stages in this study to understand the molecules responsible for odontogenic potential.

Initially, we systematically conducted RNA sequencing on developing dental epithelium and mesenchyme at E11.5 and E13.5. PCA, DEG analysis, and GO term analysis were performed using these datasets. Through qPCR examination of tissue markers of Krt14 and Vim in epithelial and mesenchymal tissues, respectively, it was confirmed that the samples were well separated. PCA further validated that the biological replicates for each group were categorized.

GO term analysis revealed the cell membrane and extracellular region as being overrepresented at both E11.5 and E13.5. It can be inferred that both the extracellular region and the cell membrane are required for orchestrating tooth formation in both stages. Previous studies have demonstrated that extracellular matrix proteins such as integrins participate in the late stages of odontogenesis, including morphogenesis and biomineralization [35, 36]. However, our results reveal that proteins in the extracellular regions are also involved in the early stages of odontogenesis and might be critical regulators of odontogenic competence and even dental stem cell commitment.

In addition to providing structural support, the ECM is now believed to elicit a cellular response by mediating various signal transduction pathways involved in development and organogenesis. The ECM contains secreted molecules that comprise the cell microenvironment and proteins that act as bridges for specific binding between the ECM and cell membrane. PGs and GAGs comprise vital parts of the ECM and are part of a finely regulated signalling network. PGs have been demonstrated to be highly involved in tooth formation. Studies have mostly focused on their roles in the late stages of tooth development, such as matrix deposition and biomineralization [16, 37]. Moreover, in our previous study, disruption of proteoglycans in the dental epithelium led to disturbed dental epithelial stem cell homeostasis due to decreased FGFR2b signalling. Although these studies have underscored the roles of PGs in the late stages of tooth formation, the role of PGs in early odontogenesis, such as tooth initiation and early morphogenesis, has not been sufficient studied.

To this end, we comprehensively mapped the proteoglycan profiles in tooth development through RNA sequencing. We identified proteoglycans with distinct expression patterns that coincide with the odontogenic shift, suggesting their potential role in early tooth development.

Interestingly, we found that some differentially expressed proteoglycans have not been described in the context of tooth formation. Most proteoglycans showed significantly higher expression levels in both the oral epithelium and mesenchyme as development progressed. Notably, several proteoglycans showed odontogenic competence in these two tissue compartments.

Syndecans are ubiquitously expressed on the cell surface of eukaryotic cells. They contain heparan sulfate side chains that provide a structural basis for syndecans to function as coreceptors for molecules such as growth factors and morphogens [38]. Within the syndecan family, our analysis found that Sdc2 was upregulated in the oral epithelium prior to the bud stage. At E13.5, the transcript level of Sdc2 was higher in the mesenchyme than in the oral epithelium. Previous studies have revealed that Sdc2 shows abundant expression in the underlying dental mesenchyme at E12.5 and E13.5 and then shifts to the stratum intermedium and inner dental epithelium at cap stages [8]. This finding indicated that Sdc2 showed odontogenic potential in the dental mesenchyme at E12.5 and E13.5 and that its expression was induced in the dental epithelium to promote further differentiation of cells within the inner dental epithelium. In human tooth development, Kero et al. found that Sdc2 was expressed on the epithelial side of the epithelial-mesenchymal interface at the late bud stage and cap stage [6]. This finding further suggested that Sdc2 might be involved in the mutual induction between odontoblasts and ameloblasts. K et al. found that Sdc2 expression might be enhanced with hard tissue formation in the late stages of tooth formation [39]. These results suggest that Sdc2 might be an essential mediator of the epithelial-mesenchymal interaction during early odontogenesis as well as a mediator of the mutual induction between odontoblasts and ameloblasts in later stages.

Sdc4 has been identified as a critical regulator of morphogenesis that can mediate various cellular processes through signalling pathways [40, 41]. We found that Sdc4 was expressed at higher levels in the dental epithelium than in the mesenchyme at both E11.5 and E13.5. These results are in line with the results of our previous immunohistological study [8]. Detection of Sdc4 in tooth formation also revealed extended strong expression in the inner enamel epithelium and stratum intermedium in the early bell stage in humans and at the bell stage in mice [6, 42]. This result suggested that Sdc4 may exert an essential role in the signalling cascade mainly within the dental epithelium. Meanwhile, Sdc4 has also been found to positively regulate the epithelial-mesenchymal transition process via TGF-β1, suggesting it has role in epithelial-mesenchymal interactions [43]. These results demonstrated that Sdc4 participates in the early development of the dental epithelium, but whether it also plays roles in the reciprocal induction between epithelial and mesenchymal cells or regulates dental epithelial stem cell commitment awaits further investigation.

Gpc4 has been widely investigated in the development of the nervous system, whereas few studies have studied Gpc4 in odontogenesis. Gpc4 belongs to the glypican family, proteoglycans that are anchored to the cell surface and modified by multiple HS side chains. These HS-modified glypicans can bind and present ligands in the ECM to the cell surface receptors, thereby modulating signalling transduction, including WNT, BMP, and FGF signalling [44–47]. Our findings indicate that the expression pattern of Gpc4 correlates with the odontogenic competence shift. On E11.5, Gpc4 is expressed at a 2-fold higher level in the dental epithelium than in mesenchymal tissue. Then, its expression was upregulated in both tissue compartments, with a 2-fold upregulation in the dental epithelium and nearly 5-fold upregulation in the mesenchymal epithelium. This result indicates that Gpc4 induces higher expression in both tissue compartments as development progresses. Research has found that Gpc4 promotes WNT signalling from the endoderm to the mesoderm to facilitate morphogenesis in zebrafish [48]. Therefore, it is also possible that Gpc4 participates in signalling between the dental epithelium and the mesenchyme. However, in-depth investigation of the roles and mechanisms underlying Gpc4 during odontogenesis is needed.

SLRPs are a distinct family of 18 proteins with various characteristics. The common feature is that they all consist of a core protein with several central leucine-rich repeats (LRRs) substituted with GAGs. Notably, our study also detected significant differential expression of several SLRPs during odontogenesis, including Dcn, Lum and Fmod.

Decorin is widely involved in hard tissue formation during odontogenesis. Decorin and biglycan are the two most investigated SLRPs regulating cell differentiation and collagen fibrillogenesis in bone and dentin formation [17, 49]. Our study provides novel information regarding the expression of these SLRPs in the earliest stage of odontogenesis. The results from our study suggested that Dcn was expressed at relatively higher levels in the dental epithelium at the early stage of tooth formation and then expressed in the underlying mesenchyme at the early bud stage. These results are consistent with previous research using in situ hybridization to show that Dcn mRNA was expressed only in the surrounding mesenchyme at the bud stage and cap stage [37]. This result suggests that during the early stage of odontogenesis, Dcn may also be involved in the dynamic signal transduction between the dental epithelium and the surrounding mesenchyme. In glioma, decorin mostly acts as a suppressor of cancer metastasis by modulating signal transduction in pathways such as c-Met/Akt/mTOR to disrupt EMT [50, 51]. Due to the differences in tumour metastasis and normal organ development, Dcn might exert differential roles as a positive regulator of epithelial-mesenchymal interactions in normal organogenesis and a negative modulator of EMT in tumour metastasis.

Lumican belongs to another class of SLRPs and is covalently bound with KS chains. However, there are only a few investigations about its role in tooth development to date [37]. Interestingly, lumican has been found to be involved in cellular processes associated with epithelial-to-mesenchymal transition (EMT) in tumorigenesis and in morphogenesis in normal multicellular organism development through multiple signalling pathways [52]. In lung development, Lum and Dcn are expressed at the epithelium-mesenchymal interface [53]. Our results found relatively high expression of Lum in the dental epithelium on E11.5 and then a significant increase in mesenchymal expression on E13.5. Distinct localization in lung development and the dynamic expression profile in early odontogenesis both suggest that Lum might regulate epithelial-mesenchymal interactions.

Notably, fibromodulin, another member of the SLPRs bearing KS chains, belongs to the same subclass as lumican and is structurally homologous to lumican. Studies have shown that they share similar collagen binding sites and participate in different stages of collagen formation [54]. In contrast to the expression pattern of Lum, the transcript level of Fmod was markedly higher in the dental epithelium at both timepoints in the current study. Previous studies have identified Fmod in the outer enamel epithelium at E14.5 [55] and in the stratum intermedium alone at P0 [16]. Together, these results indicate that Fmod might be an important regulator in the dental epithelium during tooth formation.

It is worth noting that most proteoglycans were expressed at higher levels in both dental epithelium and mesenchymal tissue as development progressed. It can be inferred that proteoglycans are not expressed following the “on” and “off” pattern. This result also suggested the possibility that the functions of proteoglycans in the context of odontogenesis are not solely determined by their expression level but might also be influenced by various posttranslational modifications, particularly sulfation and epimerization [56, 57]. These modifications provide great functional diversity for proteoglycans and modulate ligand-binding affinity in a spatiotemporal-specific way [58].

Thus, we also analysed the expression pattern of biosynthetic enzymes responsible for both the synthesis and modification of proteoglycans. Although most enzymes are differentially expressed between the epithelium and mesenchyme, the differences are not statistically significant. Because these enzymes are responsible for the biosynthesis of every proteoglycan, it is reasonable that there might only be slight differences observed in their total expression level.

A proper level of GAGs is critical for the normal organogenesis of lung, kidney and gland organogenesis, which requires epithelial-mesenchymal interactions [59, 60]. Fam20b is responsible for the 2-O-phosphorylation of xylose in the glycosaminoglycan-protein linkage region of proteoglycans, thereby regulating the abundance of mature GAG chains. From our results, Fam20b was more highly expressed in the dental epithelium at both stages. This finding matches the result of Wu et al., who revealed that epithelial-derived GAGs are critical for regulating FGF-SHH signalling between dental epithelial and mesenchymal compartments [18]. It is worth noting that such a phenotype is only observed when inactivating *Fam20b* in the dental epithelium. Conversely, no overt phenotype in tooth development was found in *Wnt1*^*Cre/*+^;*Fam20B*^*fl/fl*^ mice [61], suggesting temporal-spatial roles of GAGs in the epithelium during early odontogenesis. Furthermore, with cell lineage tracing technology, they found that the deletion of GAGs hindered PGs in assisting the diffusion of extracellular ligand, resulting in unrestricted FGF10 binding to FGFR2B. This overactivation of FGF10/FGFR2B signalling interfered with the homeostasis of *Sox2*^+^ stem cells and promoted their self-renewal. Under normal conditions, *Sox2*^+^ stem cells only localized in the labial cervical region after the completion of tooth germ development as stem cell reserves. However, in GAG-deficient mice, a group of cells with high expression of Sox2 still existed on the lingual side of the normal incisor (the area where the supernumerary teeth appeared), suggesting that ablation of GAGs may interfere with the cell fate determination of *Sox2*^+^ stem cells in the dental lamina and activate their potential for odontogenesis [18].

Previous studies have shown that HS regulates organogenesis involving epithelial-mesenchymal interactions by participating in FGF-SHH signalling. Deletion of Ext1 in lung epithelial cells led to a reduced number of mesenchymal cells with dispersed distribution and formed irregularly arranged nodes, suggesting that HS chains in the epithelium were responsible for the transduction of proper SHH signals to the mesenchyme to ensure downstream signalling in mesenchymal tissue [60]. Notably, in our study, Ext1 exhibited 1.2-fold higher expression in the epithelium on E11.5 and 1.2-fold higher expression in the mesenchyme on E13.5. Such a dynamic expression switch in accordance with odontogenic competence suggested that Ext1-catalysed HS might be critical for the epithelial-mesenchymal interaction.

Moreover, GAG epitopes and sulfation patterns affect the binding specificity and affinity of PGs with various biologically active molecules, such as growth factors and morphogens, and thereby regulate various signalling pathways that are highly relevant to early odontogenesis, such as FGF, BMP, WNT and SHH signalling.

Fgf8 is one of the first markers of tooth initiation, and fgf8-expressing epithelial rosettes migrate towards the initiating tooth bud, where they contribute essential cell mass for tooth development [62]. Hs2st and Hs6st regulate the formation of the fgf8 gradient by modulating its amplitude in the developing brain [63]. In our results, on E11.5, Hs2st1 was expressed at nearly the same level in the epithelium and mesenchymal tissue, and Hs6st1 was expressed at a 2-fold higher level in the epithelium than in the mesenchymal tissue. This result indicates that during tooth initiation, Hs6st1 might be critical for maintaining the fgf8 gradient for normal epithelial rosette formation in the tooth bud.

Wnt/β-catenin signalling is also required for early tooth morphogenesis, both in the epithelium and the mesenchyme. N-sulfation-rich HS clusters are required for Wnt/β-catenin signalling and are internalized and associated with the Frizzled/Wnt/LRP6 signalosome [64]. During tooth initiation on E11.5, Ndst1 and Ndst2 were expressed at almost the same level in both the epithelium and mesenchyme. This result suggested that N-sulfation might be involved in forming normal Wnt signalosomes in both tissue compartments to build reciprocal signalling induction critical for early odontogenesis.

FGF signalling via FGFR2 in the epithelium is crucial for cell proliferation activity during tooth development. FGFR2b is expressed in the dental epithelium, and its ligand fgf10 is expressed in the dental mesenchyme. 3-O-HS binds FGFR2b and stabilizes FGF10/FGFR2b complexes in a receptor- and growth factor-specific manner and induces rapid autocrine feedback to increase the cellular biosynthesis of 3-O-HS to promote epithelial progenitor expansion in foetal salivary glands [65]. Our data revealed a complex expression pattern of the 5 isoforms of Hs3st isoforms. Hs3st2, Hs3st4, and Hs3st5 were expressed at low levels (FPKM < 1) and therefore considered not to be expressed in early odontogenesis. Hs3st1 showed 1.2-fold higher expression in the epithelium on E11.5, whereas on E13.5, its expression level in the mesenchyme was 2-fold higher than that in the epithelium. Hs3st3a1 and Hs3st3b1 were both expressed at higher levels in the mesenchymal cells at both stages. This result indicated that different Hs3st isoforms might be responsible for sulfation in different compartments. Hs3st3a1 and Hs3st3b1 might produce 3-O-HS mostly in the dental mesenchyme to regulate or bind fgf10. Meanwhile, the dynamic expression of HS3st1 in accordance with the odontogenic competence shift indicated possible transduction of FGF10/FGFR2b signalling from the epithelium to the mesenchymal tissue. Our research provides fundamental data to understand GAG sulfation and its potential roles in signal transduction during early odontogenesis.

The current research took an important step in understanding the transcript level of PGs and their biosynthetic enzymes during odontogenesis. Proteoglycans and the GAG chains act as novel and critical signalling regulators and might play roles in the epithelium-mesenchymal interaction in early odontogenesis. Moreover, various sulfation codes have already been demonstrated to have regulatory functions by maintaining gradient diffusion and the formation and stabilization of signalling complexes in odontogenesis-related signalling. However, due to the complexity of GAG biosynthesis, the transcript levels might not comprehensively reflect the roles of GAGs. Therefore, it is increasingly urgent to apply robust and sensitive glycoproteomic analytical methods combined with bioinformatic approaches to identify potential novel proteoglycans and sulfation codes during early odontogenesis [66–68].

Furthermore, in-depth exploration of proteoglycans and their underlying mechanisms during odontogenesis requires robust evidence from animal models. Understanding how proteoglycans participate during this complicated epithelial-mesenchymal interaction will require a variety of conditional knockout animal models due to their highly spatiotemporal-dependent functions.

Previous studies mainly focused on the later stages in which proteoglycans, including perlecan, decorin, biglycan, and fibromodulin, have been demonstrated to be critical regulators in enamel and dentin matrix mineralization [16, 17, 69]. Dentin hypomineralization was observed in *Dcn*^*−/−*^ mice, *Bgn*^*−/−*^ mice and *Fmod*^*−/−*^ mice. However, these proteoglycans seemed to exert different functions in enamel formation; *Bgn*^*−/−*^ mice were demonstrated to have increased enamel formation, whereas *Dcn*^*−/−*^mice were found to have delayed enamel formation [17, 69]. However, no single proteoglycan knockout mouse exhibited a significant phenotype in the early stage. Thus, this study provides useful information for seeking potential proteoglycans during the early stages of odontogenesis.

Moreover, there is currently little evidence concerning whether proteoglycans function in the epithelial or mesenchymal compartments. Current evidence has shown that perlecan is critical for tooth morphology, as shown by transgenic mice that overexpress perlecan in epithelial cells [70]. The roles of other types of proteoglycans and whether they participate in the epithelial or mesenchymal compartments remain unclear. Furthermore, as proteoglycans and their GAGs have been identified as essential mediators of dental epithelial stem cells in *K14*^*Cre/*+^*;Fam20B*^*fl/fl*^ mice [18], further studies are needed to clarify the specific type of proteoglycans that participate in odontogenesis. And the discrepancy between *K14*^*Cre/*+^*;Fam20B*^*fl/fl*^ mice and *Wnt*^*Cre/*+^*;Fam20B*^*fl/fl*^ mice indicated that potential proteoglycans in the dental epithelium but not in the mesenchyme are critical for modulating the odontogenesis of supernumerary teeth. Thus, it is essential to identify proteoglycans expression in the dental epithelium and mesenchyme respectively during early odontogenesis. Our data provide clues in seeking potential types of proteoglycans and GAGs as well as their potential roles in the dental epithelial and mesenchymal regions during early odontogenesis. Future studies with more well-designed animal models should focus on elucidating their functions in a tissue-specific and stage-specific manner.

## Conclusion

This study of RNA-Seq data presented a new genome-wide identification of potential genes in odontogenesis research. Our findings indicate that proteoglycans show spatially and temporally distinct expression during tooth development. The proteoglycans identified in the current study can help shed light on the molecular mechanism underlying the epithelium-mesenchyme interaction during odontogenesis. Changes in proteoglycans may play pivotal roles in the regulation of numerous signal transduction pathways that finely guide tooth formation. In-depth experiments such as gain-of-function and loss-of-function studies using the candidate switch genes identified in this study should be performed to further elucidate the roles of proteoglycans during tooth formation.

## Supplementary Information

**Additional file 1.**

**Additional file 2.** GSEA analysis of DEGs from both timepoints. A GSEA of DEGs of E11.5. B GSEA of DEGs of E13.5. (DEGs, differentially expressed genes).

**Additional file 3.** Heatmap of proteoglycan and the biosynthetic enzymes at E11.5 and E13.5 in both the dental epithelium and mesenchyme. A Heatmap of proteoglycans at E11.5 and E13.5 in both the dental epithelium and mesenchyme. B Heatmap of proteoglycan biosynthetic enzymes at E11.5 and E13.5 in both the dental epithelium and mesenchyme.

## Data Availability

The datasets generated during the current study are available in the NCBI Gene Expression Omnibus (GEO) datasets with the accession number < GSE209968 > (https://www.ncbi.nlm.nih.gov/geo/query/acc.cgi?acc=GSE209968).

## Abbreviations

DEGs: Differentially expressed genes
GO: Gene Ontology
KEGG: Kyoto Encyclopedia of Genes and Genomes
FPKM: Fragments Per Kilobase per Million
GAGs: Glycosaminoglycan chains
HS: Heparan sulfate
CS: Chondroitin sulfate
DS: Dermatan sulfate
KS: Keratan sulfate
