# Supplementary material for: Genome-wide identification of potential odontogenic genes involved in the dental epithelium-mesenchymal interaction during early odontogenesis
Source: BMC Genomics. 2023 Apr 3;24:163. doi: 10.1186/s12864-023-09140-8 (PMC10069120; doi:10.1186/s12864-023-09140-8)
Supplement: Supplementary file 1 — Additional file 1. [file 12864_2023_9140_MOESM1_ESM.docx]

| **Sample** | **Raw Data** |  | **Valid Data** |  | **Valid Ratio(reads)** | **Q20%** | **Q30%** | **GC content%** |
| --- | --- | --- | --- | --- | --- | --- | --- | --- |
|  | Read | Base | Read | Base |  |  |  |  |
| E11_E | 55970694 | 8.40G | 54596928 | 8.19G | 97.55 | 99.89 | 97.18 | 48.5 |
| E11_E_2 | 51974732 | 7.80G | 50010188 | 7.50G | 96.22 | 99.93 | 97.8 | 49.5 |
| E11_E_3 | 51383626 | 7.71G | 49440246 | 7.42G | 96.22 | 99.93 | 97.74 | 49.5 |
| E11_M | 55774578 | 8.37G | 54377822 | 8.16G | 97.5 | 99.89 | 97.07 | 48.5 |
| E11_M_2 | 53017446 | 7.95G | 51152542 | 7.67G | 96.48 | 99.93 | 97.8 | 49.5 |
| E11_M_3 | 51311532 | 7.70G | 49517334 | 7.43G | 96.5 | 99.92 | 97.59 | 49.5 |
| E13_E | 54896750 | 8.23G | 53506734 | 8.03G | 97.47 | 99.89 | 97.2 | 49 |
| E13_E_2 | 55318776 | 8.30G | 53424274 | 8.01G | 96.58 | 99.93 | 97.82 | 49.5 |
| E13_E_3 | 51216198 | 7.68G | 49405032 | 7.41G | 96.46 | 99.93 | 97.83 | 49 |
| E13_M | 53783708 | 8.07G | 52427656 | 7.86G | 97.48 | 99.89 | 96.97 | 49 |
| E13_M_2 | 51423474 | 7.71G | 49541466 | 7.43G | 96.34 | 99.93 | 97.77 | 49.5 |
| E13_M_3 | 51944588 | 7.79G | 50172160 | 7.53G | 96.59 | 99.93 | 97.88 | 49.5 |

Supplement file1. Summary statistics for sequence quality control and mapped data of sample
