# Supplementary figures and images for: Genome-wide identification of potential odontogenic genes involved in the dental epithelium-mesenchymal interaction during early odontogenesis
Source: BMC Genomics. 2023 Apr 3;24:163. doi: 10.1186/s12864-023-09140-8 (PMC10069120; doi:10.1186/s12864-023-09140-8)

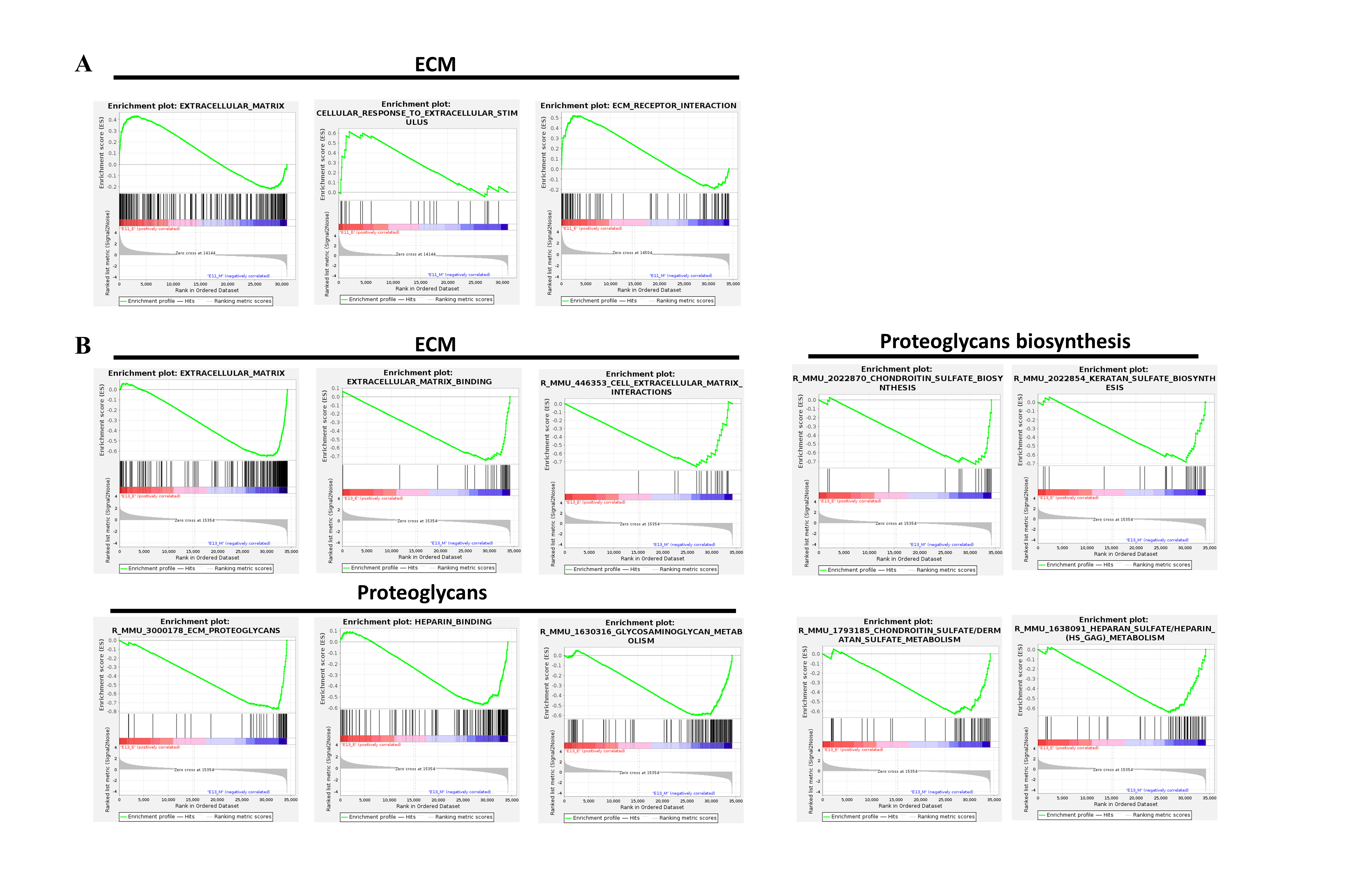

Supplement: Supplementary file 2 — Additional file 2. GSEA analysis of DEGs from both timepoints. A GSEA of DEGs of E11.5. B GSEA of DEGs of E13.5. (DEGs, differentially expressed genes). [file 12864_2023_9140_MOESM2_ESM.png]

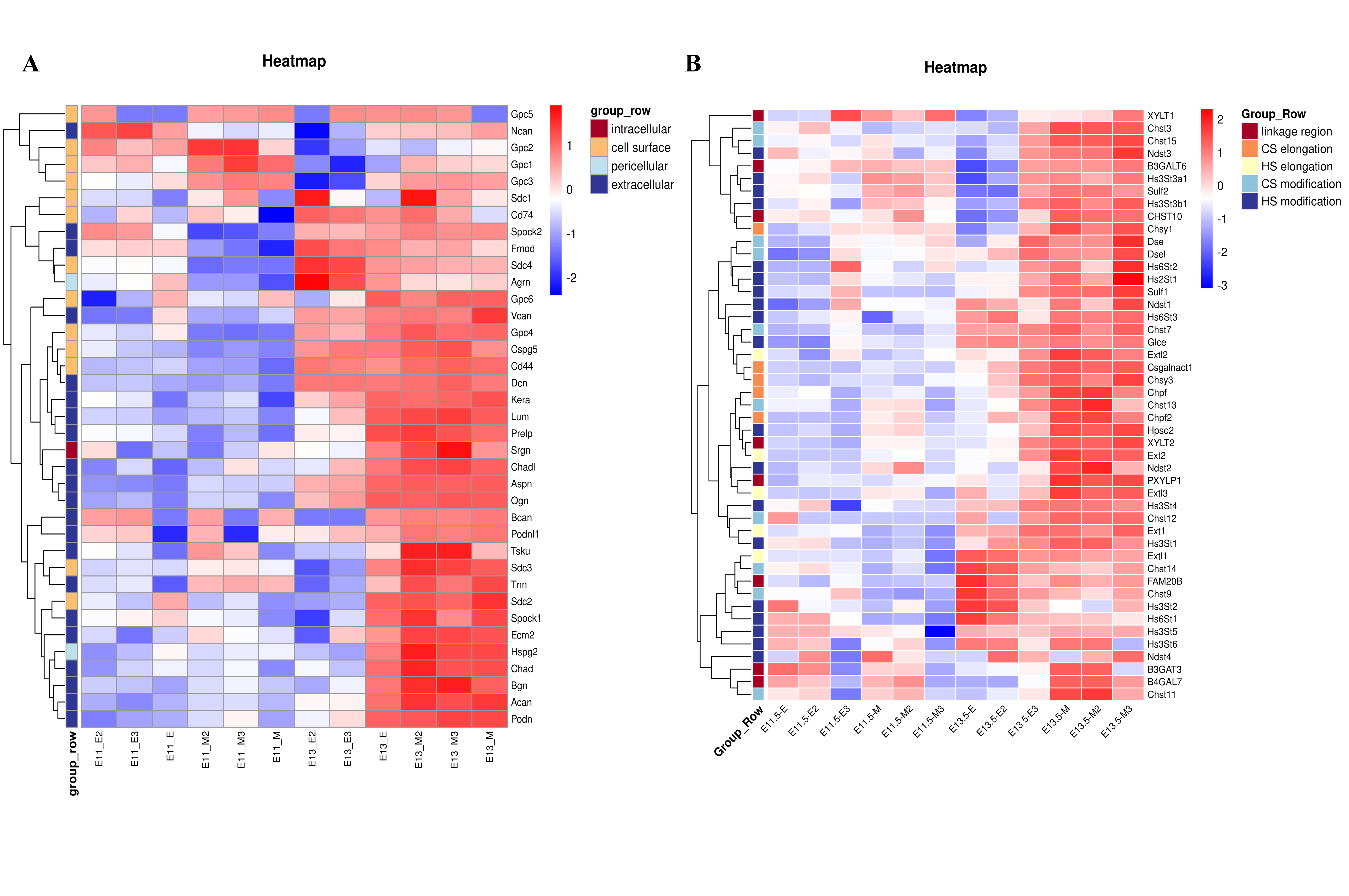

Supplement: Supplementary file 3 — Additional file 3. Heatmap of proteoglycan and the biosynthetic enzymes at E11.5 and E13.5 in both the dental epithelium and mesenchyme. A Heatmap of proteoglycans at E11.5 and E13.5 in both the dental epithelium and mesenchyme. B Heatmap of proteoglycan biosynthetic enzymes at E11.5 and E13.5 in both the dental epithelium and mesenchyme. [file 12864_2023_9140_MOESM3_ESM.png]
